# Psychiatric symptoms in huntington’s disease

**DOI:** 10.1192/j.eurpsy.2021.682

**Published:** 2021-08-13

**Authors:** C. Peixoto, D. Rego, M. Bicho, J. Coelho, H. Medeiros

**Affiliations:** Psychiatry, Hospital do Divino Espírito Santo de Ponta Delgada, E.P.E., Ponta Delgada, Portugal

**Keywords:** Huntington’s disease, Psychiatric symptoms, Depression, psychiatric comorbidity

## Abstract

**Introduction:**

Huntington’s disease (HD) is an autosomal dominant neurodegenerative disorder, that typically manifests in adulthood, clinically characterized by progressive motor, cognitive and psychiatric/behavioural symptoms. Psychiatric symptoms are common in HD. The presentation of these symptoms is highly variable, and their course does not correlate with motor or cognitive disease progression. Psychiatric symptoms often precede motor onset by many years.

**Objectives:**

The authors intend to review the literature the most frequent psychiatric disorders in patients with HD.

**Methods:**

Non-systematic review of the literature.

**Results:**

Psychiatric symptoms have been a core feature of HD. Pre-symptomatic HD patients exhibit a greater prevalence of psychiatric symptoms, particularly affective disorders. This symptoms are presenting symptoms of HD in up to half of all people. In symptomatic HD patients, it is estimated that up to 73–98% of patients will have a major psychiatric disorder or psychiatric symptoms. Psychiatric manifestations in HD include depression, irritability, apathy, anxiety, mania, perseverations, obsessions and psychosis. Cognitive changes include progressive deficits in attention, learning, executive and sensory functions, resulting in dementia. Depression, diagnosed in half of patients with HD, is the most common and earliest symptoms prior to the motor onset. There are likely multiple causes of the psychiatric symptoms, with underlying factors including a combination of neurobiological, cognitive, psychological, social and environmental factors.

**Conclusions:**

Patients with HD have high psychiatric comorbidity, that causes significant functional impairment and affect quality of life. Thus, they require a multidisciplinary approach in the recognition and treatment of psychiatric symptoms.

